# Alterations of the Ileal and Colonic Mucosal Microbiota in Canine Chronic Enteropathies

**DOI:** 10.1371/journal.pone.0147321

**Published:** 2016-02-03

**Authors:** Eric Cassmann, Robin White, Todd Atherly, Chong Wang, Yaxuan Sun, Samir Khoda, Curtis Mosher, Mark Ackermann, Albert Jergens

**Affiliations:** 1 Department of Veterinary Clinical Sciences, College of Veterinary Medicine, Iowa State University, Ames, Iowa, United States of America; 2 USDA-ARS, Ames, Iowa, United States of America; 3 Department of Veterinary Diagnostic and Production Animal Medicine, College of Veterinary Medicine, Iowa State University, Ames, Iowa, United States of America; 4 Department of Genetics, Development & Cell Biology, College of Liberal Arts and Sciences, Iowa State University, Ames, Iowa, United States of America; 5 Department of Veterinary Pathology, College of Veterinary Medicine, Iowa State University, Ames, Iowa, United States of America; University of Minnesota, UNITED STATES

## Abstract

**Background:**

The intestinal microbiota is increasingly linked to the pathogenesis of chronic enteropathies (CE) in dogs. While imbalances in duodenal and fecal microbial communities have been associated with mucosal inflammation, relatively little is known about alterations in mucosal bacteria seen with CE involving the ileum and colon.

**Aim:**

To investigate the composition and spatial organization of mucosal microbiota in dogs with CE and controls.

**Methods:**

Tissue sections from endoscopic biopsies of the ileum and colon from 19 dogs with inflammatory bowel disease (IBD), 6 dogs with granulomatous colitis (GC), 12 dogs with intestinal neoplasia, and 15 controls were studied by fluorescence in situ hybridization (FISH) on a quantifiable basis.

**Results:**

The ileal and colonic mucosa of healthy dogs and dogs with CE is predominantly colonized by bacteria localized to free and adherent mucus compartments. CE dogs harbored more (*P* < 0.05) mucosal bacteria belonging to the *Clostridium-coccoides/Eubacterium rectale* group, *Bacteroides*, *Enterobacteriaceae*, and *Escherichia coli* versus controls. Within the CE group, IBD dogs had increased (*P* < 0.05) *Enterobacteriacea*e and *E*. *coli* bacteria attached onto surface epithelia or invading within the intestinal mucosa. Bacterial invasion with *E*. *coli* was observed in the ileal and colonic mucosa of dogs with GC (*P* < 0.05). Dogs with intestinal neoplasia had increased (*P* < 0.05) adherent (total bacteria, *Enterobacteriaceae*, *E*. *coli*) and invasive (*Enterobacteriaceae*, *E*. *coli*, and *Bacteroides*) bacteria in biopsy specimens. Increased numbers of total bacteria adherent to the colonic mucosa were associated with clinical disease severity in IBD dogs (*P* < 0.05).

**Conclusion:**

Pathogenic events in canine CE are associated with different populations of the ileal and colonic mucosal microbiota.

## Introduction

Chronic enteropathies (CE), including idiopathic inflammatory bowel disease (IBD), food responsive enteropathy (FRE), and alimentary neoplasia are common causes for persistent or recurrent gastrointestinal (GI) signs in dogs [1–3]. While the exact etiology for these disorders remains unknown, accumulating evidence suggests a pivotal role for the intestinal microbiota in disease pathogenesis. Recent studies using 16S rRNA molecular analyses (ie, high throughput [HTP] sequencing and fluorescence in situ hybridization [FISH]) have shown that canine CE is associated with altered microbial composition characterized by reduced diversity and/or selective enrichment with individual bacterial species [4–7].

Canine CE may be multifocal (ie, spectrum of patchy to diffuse mucosal involvement) in its distribution which supports the acquisition of biopsy samples from multiple intestinal regions [8]. Moreover, separate studies investigating dogs with CE indicate that histopathologic findings of endoscopically-obtained duodenal, ileal, and colonic biopsies may vary significantly within the same dog [9]. While bacterial 16S rRNA–based methods have shown an association between altered microbial composition and duodenal inflammation, relatively little is known about alterations in mucosal bacteria seen with CE involving the ileum and colon [10,11].

The aim of the present study was to utilize FISH techniques to identify and compare the mucosal microbiota of concurrent endoscopically-obtained ileal and colonic biopsies from dogs diagnosed with CE. In addition, we examined the potential relationship of mucosal bacteria, clinical disease activity, and histopathology which might be associated with different causes of canine CE.

## Materials and Methods

### Ethical animal use

The collection and analysis of intestinal biopsies obtained endoscopically from dogs with CE were previously approved by the Iowa State University Institutional Animal Care and Use Committee. Written informed consent was obtained from all owners of dogs enrolled in separate trials (IACUC Log numbers: 1-11-7061-K, 12-11-7269-K).

### Canine CE disease groups and controls

Four dog groups were studied with groups 1–3 representing CE dogs while group 4 dogs served as controls. The diagnostic evaluation in all CE dogs consisted of extensive medical histories taken over multiple clinical examinations, hematological and serum biochemistry analyses, urinalysis, fecal examinations for parasites, diagnostic imaging (including abdominal sonography), and histopathologic examination of mucosal biopsy specimens. In some dogs, samples were additionally collected for a measurement of serum concentration of pancreatic-lipase immunoreactivity (cPLI), trypsin-like immunoreactivity (cTLI), cobalamin, and folate concentrations. None of the CE dogs had received glucocorticoid or antibiotic therapy within 3 weeks of being diagnosed with CE. Clinical disease activity was calculated for all dogs using the **C**anine **I**nflammatory **B**owel **D**isease **A**ctivity **I**ndex (CIBDAI) [3].

Group 1 comprised a group of 19 dogs diagnosed with idiopathic IBD according to previously published criteria [1–3]. The enrollment criteria included persistent (> 3 weeks duration) GI signs, failure to respond to appropriate dietary (elimination diet fed exclusively for at least 3 weeks) and antibiotic (metronidazole or tylosin administered exclusively for 14 days or more) trials, failure to document other causes for gastroenteritis by thorough diagnostic testing, and histopathologic evidence of mucosal inflammation observed in endoscopic biopsies.

Group 2 dogs (n = 6) were diagnosed with granulomatous colitis using traditional IBD diagnostic criteria coupled with specialized immunohistochemical (PAS Stain) and/or molecular (FISH) confirmation of attaching/invasive *Esherichia coli* bacteria identified within colonic mucosal biopsy specimens [10,11].

Group 3 dogs (n = 12) were definitively diagnosed with malignant alimentary tract neoplasia based on microscopic evidence of neoplastic cells infiltrating within the lamina propria of endoscopically-obtained samples of ileal and/or colonic mucosa. Individual tumor types included adenocarcinoma (n = 9) and lymphosarcoma (n = 3).

Group 4 dogs served as controls and were comprised of 13 young (<2 years old) healthy laboratory-reared beagles and 2 young mongrel dogs. Each of these dogs was free of GI signs over several months preceding diagnostic evaluation. Moreover, control dogs were judged to be healthy on the basis of normal results on physical examination, hematological and serum biochemical analysis, urinalysis, multiple fecal examinations, and dirofilaria antigen assay.

### Intestinal biopsy and histopathologic examination

All dogs had ileoscopy/colonoscopy performed for the collection of distal intestinal mucosal biopsy specimens. Dogs were prepared for colonoscopy by withholding food overnight and administering an oral colonic electrolyte lavage solution, twice, at a dosage of 20 ml/kg. One or two tepid water enemas (20 ml/kg) were performed in the morning prior to endoscopic examination. Prior to endoscopy, the endoscope and biopsy forceps were thoroughly cleaned and sterilized using an activated aldehyde solution and gas sterilization, respectively. Multiple (12–15 samples from the colon; 5–7 samples from the ileum) endoscopic biopsies were obtained and fixed in 10% neutral buffered formalin and then paraffin embedded for use in histopathology, using HE stains, and for FISH. Ileal mucosal biopsies were collected along a 10 cm segment of distal ileum while colonic mucosal biopsies were obtained from each of the ascending, transverse, and descending colonic regions. Lastly, mass lesions involving the distal descending colon and/or rectum were biopsied directly.

Histopathologic examination of endoscopic paraffin-embedded tissue sections was performed by a single pathologist (MA) blinded as to each dog’s history and clinical course. Tissues were graded for severity of intestinal mucosal inflammation using simplified WSAVA histopathologic criteria [12].

### Fluorescence in situ hybridization (FISH)

The formalin-fixed embedded histopathological tissue sections were mounted on glass slides and evaluated by fluorescence in situ hybridization (FISH) as previously described [13–15]. In brief, paraffin-embedded tissue specimens were deparaffinized using an automated system by passage through xylene (3 x 10 min), 100% alcohol (2 x 5 min), 95% ethanol (5 min), and finally 70% ethanol (5 min). The slides were next rapidly transported in deionized water to the DNA testing laboratory where they were air dried prior to hybridization. FISH probes 5’-labeled with either Cy-3 or FITC (Life Sciences) were reconstituted with DNAse-free water and diluted to a working concentration of 5 ng/μL (Table 1).

**Table 1:** Probes used for fluorescence in situ hybridization. Probes used for in situ bacterial identification.

Probe	Sequence (5’ → 3’)	Target	Reference
Eub338	GCT GCC TCC CGT AGG AGT	Eubacteria	Amann (1990)
Erec482	GCT TCT TAG TCA RGT ACC G	*Eubacterium rectale-Clostridium coccoides*	Frank (1998)
Ebac1790	CGT GTT TGC ACA GTG CTG	Enterobacteriaceae	Poulsen (1994)
Ec	GCA AAG GTA TTA ACT TTA CTC CC	*E*. *coli* and *Shigella*	McGregor (1996)
Bac303	CCA ATG TGG GGG ACC TT	*Bacteroides* spp.	Manz (1996)
Hel717	AGG TCG CCT TCG CAA TGA GTA	*Helicobacter* spp.	Jergens (2010)
Non-Eub338	ACT CCT ACG GGA GGC AGC	Irrelevant probe	Janeczko (2008)

For total bacterial counts EUB338-FITC was used. For other analyses, specific probes targeting Clostridium, *Bacteroides-Prevotella*, *Enterobacteriaceae*, *E coli*, Lactobacilli, and Helicobacter were labeled with Cy-3 and were applied simultaneously with the universal bacterial probe Eub338-FITC [16–20]. This battery of probes was selected to identify specific bacterial groups and individual bacterial species previously shown to be relevant in the pathogenesis of canine CE [4–7]. Tissue sections were bathed in 30 μL of DNA–probe mix in a hybridization chamber maintained at 54°C overnight (12 h). Washing was performed using a wash buffer (hybridization buffer without SDS), the slides were rinsed with sterile water, then allowed to air-dry, and mounted with SlowFade Gold mounting media (Life Technologies, Carlsbad, CA) and 25X25-1 cover glass (Fisher Scientific, Pittsburgh, PA).

Probe specificity was confirmed in pilot studies by combining the irrelevant probe non-Eub338-FITC with Eub338-Cy-3, and through hybridization experiments with pure isolates of Clostridia, Bacteroides, *Enterobacteriaceae*, and *E*. *coli* to screen for non-selective hybridization. Archived sections of gastric mucosa from a dog diagnosed with Helicobacter infection was used as a positive control for helicobacter FISH [14].

### In situ quantification of mucosal bacteria

The bacteria were visualized by FISH and 4,6-diamidino-2-phenylindole (DAPI) staining using a 60x Plan Apo oil objective in conjunction with an optional 1.5x multiplier lens on an Eclipse TE2000-E fluorescence microscope (Nikon Instruments Inc., Melville NY) and photographed with a CoolSnap EZ camera (Photometrics, Tuscon, AZ) controlled by MetaMorph software (Nashville, TN). Quantification was only performed when the hybridization signals were strong and could clearly distinguish intact bacteria morphologically by either 2-color (universal and bacterial specific FISH probe) or 3-color (FISH probes and DAPI stain) identification. A minimum of 4 different endoscopic biopsy specimens/organs were evaluated for their mucosal bacterial content. Bacterial quantification was performed in 10 representative fields at a final observed magnification of 600x or 900x. The ten fields included bacteria found within 4 well-defined mucosal compartments: (1) bacteria contained within the mucosa, (2) bacteria attached to the surface epithelium, (3) bacteria localized within adherent mucus, and (4) bacteria found within free mucus.

### Statistics

Data were analyzed using generalized linear mixed models for each of the colon and ileum tissues. A negative binomial distribution was used in the generalized model for the response, the number of enumerated bacteria. For each analysis, group (control, IBD, GC, or cancer), probe, mucosal compartment and their interactions were included as a fixed effect, whereas each dog was treated as a random effect. F-tests were used to test the significance of main effects and interactions. If significant overall differences were identified among levels of a factor, post-hoc pairwise comparisons were performed using t-tests with Tukey's adjustment. To test the correlation between mucosal microbiota and inflammatory indices, spearman’s rank correlation coefficients and the corresponding p values were calculated. The correlations between the summary counts of attaching and invasive bacteria and each of the severe histologic inflammation and CIBDAI scores were calculated. All analyses were performed in SAS 9.4 (SAS Institute, Cary NC) with a P-value of < 0.05 considered significant.

## Results

### Patient characteristics

Patient demographics of healthy and diseased dogs, including dietary history at the time of diagnosis, are found in Table 2. The base-line clinical characteristics in CE dogs were similar to previous reports (Table 3) [1–3]. Dogs with intestinal cancer were oldest while most IBD dogs were middle-aged (age range 1–14 years). All CE dogs exhibited variable yet chronic GI signs indicative of colitis (n = 14) or enterocolitis (n = 23). Three CE dogs had mild disease activity (including 2 dogs with IBD and 1 dog with GC); 9 dogs had moderate disease activity including 5 dogs with IBD, 1 dog with GC, and 3 dogs with neoplasia); and 12 dogs had severe disease activity (including 11 dogs with IBD and 1 dog with neoplasia) based on CIBDAI scores. Thirteen dogs with a CIBDAI score of 3 exhibited only signs of colitis. Endoscopic mucosal abnormalities involving the ileum and/or colon (ie, increased friability, granularity, and/or erosions) were observed in 94% of CE dogs. Histopathologic review of intestinal biopsy specimens showed a 37% and 63% distribution of mild versus moderate-to-severe inflammatory lesions in dogs with IBD and GC, respectively.

**Table 2:** Dog cohort demographics. Canine cohort demographics with regards to age, gender, breed, and diet at the time of endoscopic biopsy.

Cohort	Age	Gender	Breed	Diet
Healthy control	2	FS	Beagle	Purina maintenance ration
Healthy control	1	FS	Beagle	Purina maintenance ration
Healthy control	2	FS	Beagle	Purina maintenance ration
Healthy control	2	FS	Beagle	Purina maintenance ration
Healthy control	1	FS	Beagle	Purina maintenance ration
Healthy control	2	FS	Beagle	Purina maintenance ration
Healthy control	3	FS	Beagle	Purina maintenance ration
Healthy control	2	FS	Beagle	Purina maintenance ration
Healthy control	2	FS	Beagle	Purina maintenance ration
Healthy control	3	FS	Beagle	Purina maintenance ration
Healthy control	2	FS	Beagle	Purina maintenance ration
Healthy control	2	FS	Beagle	Purina maintenance ration
Healthy control	1	FS	Beagle	Purina maintenance ration
Healthy control	1	F	Mongrel	Purina maintenance ration
Healthy control	1	F	Mongrel	Purina maintenance ration
Idiopathic IBD	5	MC	Dachshund	Prescription Hill’s i/d
Idiopathic IBD	9	F	Standard Poodle	Prescription Hill’s z/d
Idiopathic IBD	2	MC	Pembroke Corgi	Prescription Hill’s i/d
Idiopathic IBD	5	MC	Weimarener	IVD Potato and Venison
Idiopathic IBD	4	FS	Miniature Schanauzer	Royal Canin Low fat Duck /Potato
Idiopathic IBD	2	MC	Jack Russel Terrier	Prescription Hill’s Duck/Potato
Idiopathic IBD	6	FS	Springer Spaniel	Prescription Hill’s i/d
Idiopathic IBD	11	FS	Boxer	Natural Balance Duck/Potato
Idiopathic IBD	2	FS	Giant Schnauzer	Purina H/A
Idiopathic IBD	1	MC	Rottweiler	Sojo’s Grain Free Diet
Idiopathic IBD	1	MC	Great Pyrenees	Homemade–Chicken/Rice
Idiopathic IBD	4	FS	Beagle	Prescription Hill’s z/d
Idiopathic IBD	1	MC	Rottweiler	Prescription Hill’s z/d
Idiopathic IBD	2	FS	Havanese	Royal Canin Low fat Duck /Potato
Idiopathic IBD	11	FS	Labrador Retriever	Prescription Hill’s d/d (Venison)
Idiopathic IBD	11	MC	West highland White Terrier	Prescription Hill’s d/d (Venison)
Idiopathic IBD	4	FS	Shih Tzu	Prescription Hill’s Duck/Potato
Idiopathic IBD	5	MC	English Bulldog	Prescription Hill’s z/d
Idiopathic IBD	9	FS	Grey Hound	Prescription Hill’s z/d
Granulomatous colitis	1	FS	Boxer	Nature’s Recipe–Chicken/Barley/Rice
Granulomatous colitis	1	FS	Boxer	Homemade–Chicken/Rice
Granulomatous colitis	1	FS	Boxer	Prescription Hill’s i/d
Granulomatous colitis	1	FS	Boxer	Purina EN Diet
Granulomatous colitis	1	FS	Boxer	Prescription Hill’s z/d
Granulomatous colitis	1	FS	Boxer	Prescription Hill’s z/d
Intestinal AdenoCA	11	MC	Brittany Spaniel	Beneful Dry Ration
Intestinal AdenoCA	8	MC	Shih Tzu	Prescription Hill’s r/d
Intestinal AdenoCA	11	MC	Shetland Sheep dog	Prescription Hill’s i/d
Intestinal AdenoCA	9	MC	Labrador Retriever	Iams Low-Residue
Intestinal AdenoCA	13	MC	Pembroke Corgi	Eagle Pack Holistic Fish
Intestinal AdenoCA	9	MC	Labrador Retriever	Prescription Hill’s i/d
Intestinal AdenoCA	7	FS	German Shorthair Pointer	Prescription Hill’s i/d
Intestinal AdenoCA	11	MC	Mongrel	Prescription Hill’s z/d
Intestinal AdenoCA	10	MC	German Shorthair Pointer	Prescription Hill’s r/d
Intestinal LSA	2	MC	Beagle	Prescription Hill’s z/d
Intestinal LSA	9	FS	Mongrel	Prescription Hill’s i/d
Intestinal LSA	6	MC	Boxer	Homemade–Chicken/Rice

**Table 3:** Clinical characteristics of dogs studied. CIBDAI = canine IBD activity index with score reported at diagnosis.

Characteristic	IBD dogs	Neoplasia dogs	GC dogs	Controls
No. of females/no. of males	10/9	2/10	6/0	15/0
Mean age (yr.)	5.0	8.2	1.0	1.8
Mean CIBDAI score	7.4	5.1	3.5	0
Dogs with signs of:				
Colitis	1	7	6	
Enterocolitis	18	5	0	

The mean age of the healthy control dogs was 1.8 years (age range 1–3 years). All dogs were females. There were no abnormalities in the results of physical examination, CBC, serum biochemical analysis, urinalysis, multiple fecal examinations, dirofilarial antigen assay, endoscopic examination, or histopathologic findings of mucosal biopsies.

### Mucosal bacteria

#### (i) Total bacteria and distribution by organ and dog group

**Fig 1:**
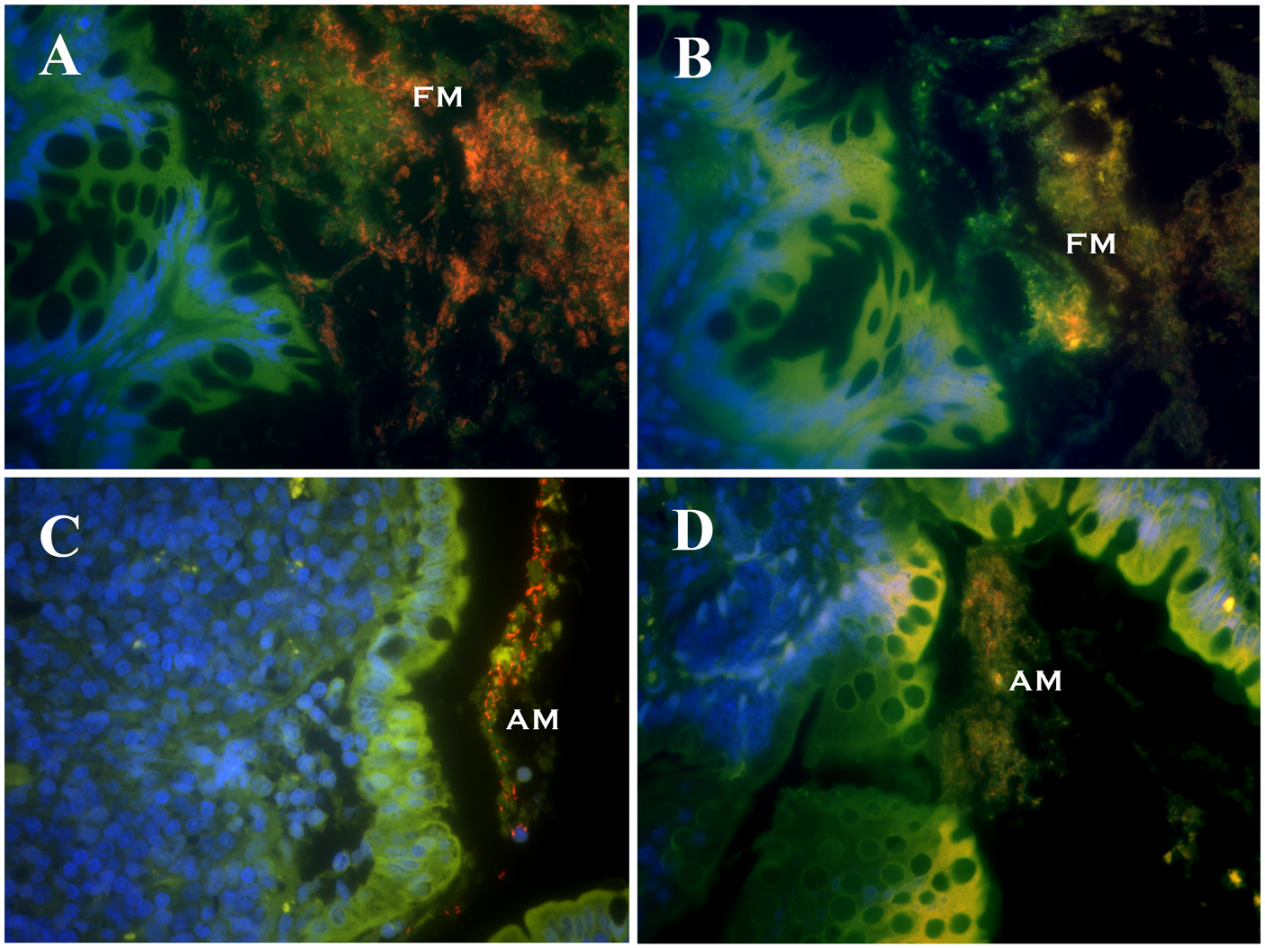
FISH of canine endoscopic biopsies. Triple color FISH identifies bacterial organisms within different mucosal compartments of endoscopic ileal and colonic biopsies obtained from healthy dogs. Panel A = colon biopsy hybridized with Cy3-Ebac1790; Panel B = colon biopsy hybridized with Cy3-Bac303; Panel C = ileum hybridized with Cy3-Ebac1790; and Panel D = ileum hybridized with Cy3-Ec (*E*. *coli*). All other bacteria that hybridize exclusively with the universal probe (Eub338-FITC) appear green. DAPI-stained colonic mucosa with goblet cells appears blue. FM = free mucus; AM = adherent mucus.

The number of total bacteria (ie, summed across all 4 mucosal compartments) identified by each probe is displayed in Tables 4 and 5. The number of Eub338-positive bacteria detected in the ileum and colon of CE dogs was not significantly (*P* > 0.05) different from healthy dogs (Fig 1). Within the different groups of CE dogs, the probes specific for *Eubacterium rectale* (Erec482), *Bacteroides* (Bac303), and members of the family *Enterobacteriaceae* and *E*. *coli* (Ebac1790 and Ec, respectively) hybridized significantly (*P* < 0.05) more total bacteria than these same bacterial sub-populations observed in healthy dogs. Dogs with intestinal neoplasia showed significantly (*P* < 0.05) increased populations of total bacteria hybridizing against probe Ebac1790 in ileal tissues, and against probes Eub338, Erec482, Ebac1790, and Bac303 in colonic tissues versus other CE dog groups. Sub-populations of bacteria (*Helicobacter* spp) which hybridized against probe Hel717 were significantly (*P* < 0.05) increased in the ileal and colonic tissues of healthy dogs.

**Table 4:** Number of ileal bacteria in healthy and diseased dogs.

	A	B	C	D
Probe	IBD (n = 19)	Neoplasia (n = 12)	GC (n = 6)	Healthy (n = 15)
**Eub338**				
Mean	8.9	18.7	14.6	12.2
Range	0–254	0–516	0–250	0–621
**Erec482**				
Mean	3.3 ^a^	4.4 ^a^	5.3 ^a^	0.4
Range	0–115	0–115	0–77	0–20
**Ebac1790**				
Mean	0.8 ^a^^,^^b^	7.2 ^a^^,^^b^	2.0 ^a^	0.7
Range	0–28	0–190	0–40	0–36
**Ec**				
Mean	1.2 ^a^	3.4	1.7 ^a^	0.5
Range	0–44	0–160	0–35	0–36
**Bac303**				
Mean	3.4 ^a^	6.8 ^a^	8.7 ^a^	0.6
Range	0–266	0–260	0–180	0–29
**Hel717**				
Mean	0	0.1 ^a^	0	1.9
Range		0–3		0–149

Data expressed as mean and range.

^a^ significant (*P* < 0.05) difference between control and CE dogs.

^b^ significant (*P* < 0.05) difference between CE groups.

**Table 5:** Number of colonic bacteria in healthy and diseased dogs.

	A	B	C	D
Probe	IBD (n = 19)	Neoplasia (n = 12)	GC (n = 6)	Healthy (n = 15)
**Eub338**				
Mean	15.1	33.2 ^b^	19.9 ^b^	16.9
Range	0–815	0–2020	0–665	0–621
**Erec482**				
Mean	9.0 ^a^^,^^b^	21.5 ^a^^,^^b^	4.5 ^b^	3.7
Range	0–397	0–1759	0–112	0–126
**Ebac1790**				
Mean	2.4 ^a^^,^^b^	8.8 ^a^^,^^b^	2.8 ^a^^,^^b^	1.1
Range	0–140	0–623	0–53	0–51
**Ec**				
Mean	2.8 ^a^^,^^b^	7.0 ^a^^,^^b^	1.9 ^a^^,^^b^	0.5
Range	0–114	0–421	0–26	0–36
**Bac303**				
Mean	9.6 ^b^	27.3 ^a^^,^^b^	5.9 ^b^	4.2
Range	0–1082	0–1872	0–210	0–225
**Hel717**				
Mean	0.4 ^a^^,^^b^	0.1 ^a^^,^^b^	0 ^a^^,^^b^	1.9
Range	0–42	0–13		0–177

Data expressed as mean and range.

^a^ significant (*P* < 0.05) difference between control and CE dogs.

^b^ significant (*P* < 0.05) difference between CE groups.

#### (ii) Disease related differences in number and distribution of mucosal microbiota

The number of bacteria identified by each probe within different mucosal compartments (ie, free mucus, adherent mucus, attached to surface epithelia, and invasive within mucosa) is displayed in Tables 6 (ileum) and 7 (colon). The number of bacteria hybridizing against probes Eub338, Erec482, Ebac1790, Ec, and Bac303 was increased (*P* < 0.05) in the free and adherent mucus compartments of CE dogs versus healthy dogs. In colonic biopsy specimens, significant (*P* < 0.05) differences in bacteria between mucosal compartments of CE dog were observed for probes hybridizing against total bacteria (Eub338), *Eubacterium rectale* (Erec482), *Enterobacteriaceae* (Ebac1790), *E*. *coli* (Ec), *Bacteroides* (Bac303) and *Helicobacter* spp (Hel717). These same differences in bacterial numbers between mucosal compartments of CE dogs were observed in ileal biopsies with the exception of bacteria hybridizing against probe Hel717. Dogs with cancer had the greatest (*P* < 0.05) number of mucus-laden bacteria versus IBD dogs and dogs diagnosed with GC.

**Table 6:** Spatial distribution of the number of ileal bacteria based on FISH.

Probe	Group	FM	AM	SE	I
**Eub338**	Control	2.2	46.4	0.2	0.1
		(0–67)	(0–621)	(0–16)	(0–2)
	IBD	4.2^b^	31.1	0.4	0.1
		(0–111)	(0–254)	(0–14)	(0–2)
	Neoplasia	17.3^a^^,^^b^	56	1.2^a^	0.1
		(0–187)	(0–516)	(0–18)	(0–2)
	GC	44.3^a^^,^^b^	13	0.9	0
		(0–250)	(0–71)	(0–20)	
**Erec482**	Control	0.1	1.5	0.1	0
		(0–15)	(0–20)	(0–4)	
	IBD	1.9^a^^,^^b^	11.2^a^	0.1	0
		(0–69)	(0–115)	(0–9)	
	Neoplasia	1.8^a^^,^^b^	15.1^a^	0.3^a^	0.1
		(0–27)	(0–115)	(0–11)	(0–4)
	GC	16.2^a^^,^^b^	5.0	0	0
		(0–77)	(0–49)		
**Ebac1790**	Control	0.1	2.6	0	0
		(0–2)	(0–36)		
	IBD	1.2^a^^,^^b^	1.9^b^	0.1^b^	0.1
		(0–27)	(0–28)	(0–3)	(0–8)
	Neoplasia	11.6^a^^,^^b^	16.5^a^^,^^b^	0.8^b^	0.1
		(0–190)	(0–119)	(0–14)	(0–3)
	GC	4.5^a^	3.3	0	0
		(0–40)	(0–32)		
**Ec**	Control	0	1.9	0	0
			(0–36)		
	IBD	0.6^b^	4.1	0.1^b^	0.1^b^
		(0–23)	(0–44)	(0–3)	(0–3)
	Neoplasia	8.1^b^	5.2	0.3^b^	0.1
		(0–160)	(0–40)	(0–4)	(0–3)
	GC	4.9^b^	1.6	0	0.4^b^
		(0–35)	(0–17)		(0–10)
**Bac303**	Control	1.0	1.0	0.1	0
		(0–28)	(0–29)	(0–6)	
	IBD	3.3^a^^,^^b^	10.0^a^	0.1	0.1
		(0–77)	(0–266)	(0–8)	(0–3)
	Neoplasia	13.0^a^^,^^b^	13.8^a^	0.4	0
		(0–260)	(0–119)	(0–5)	
	GC	26.3^a^^,^^b^	8.1^a^	0.2	0
		(0–180)	(0–66)	(0–6)	
**Hel717**	Control	0	7.8	0	0
			(0–149)		
	IBD	0	0	0	0
	Neoplasia	0	0.4^a^	0	0
			(0–3)		
	GC	0	0	0	0

Data expressed as mean and range.

^a^ significant (*P* < 0.05) difference between control and CE dogs.

^b^ significant (*P* < 0.05) difference between CE groups.

**Table 7:** Spatial distribution of the number of colonic bacteria based on FISH.

Probe	Group	FM	AM	SE	I
**Eub338**	Control	10.1	55.4	2.0	0.2
		(0–240)	(0–621)	(0–40)	(0–6)
	IBD	22.5^b^	36.2	1.4	0.5^a^
		(0–815)	(0–411)	(0–91)	(0–30)
	Neoplasia	91.2^a^^,^^b^	37.8	2.9^a^^,^^b^	0.8
		(0–2020)	(0–516)	(0–49)	(0–14)
	GC	63.6^a^	14.7^a^	0.8^b^	0.3
		(0–665)	(0–121)	(0–10)	(0–10)
**Erec482**	Control	4.5	9.8	0.5	0.1
		(0–126)	(0–109)	(0–20)	(0–3)
	IBD	8.2^b^	27.8^a^^,^^b^	0.4^b^	0.1
		(0–397)	(0–270)	(0–20)	(0–8)
	Neoplasia	65.8^a^^,^^b^	18.8^b^	2.3^a^^,^^b^	0.2
		(0–1759)	(0–320)	(0–27)	(0–6)
	GC	12.8^b^	4.9^b^	0.2^b^	0
		(0–112)	(0–50)	(0–6)	
**Ebac1790**	Control	0.4	4.2	0.1	0
		(0–30)	(0–51)	(0–3)	
	IBD	4.1^a^^,^^b^	4.9	0.3^a^	0.1
		(0–140)	(0–45)	(0–30)	(0–7)
	Neoplasia	29.2^a^^,^^b^	5.6	0.3^a^	0.1^b^
		(0–623)	(0–49)	(0–14)	(0–2)
	GC	5.5^a^^,^^b^	5.0	0	0.5^b^
		(0–53)	(0–26)		(0–8)
**Ec**	Control	0.2	2.0	0	0
		(0–9)	(0–36)		
	IBD	3.0^a^^,^^b^	8.0^a^	0.1^b^	0.1
		(0–100)	(0–114)	(0–3)	(0–1)
	Neoplasia	20.6^a^^,^^b^	7.0^a^	0.4^a^^,^^b^	0.1^b^
		(0–421)	(0–193)	(0–10)	(0–10)
	GC	1.6^a^^,^^b^	3.9	0.1	2.2^b^
		(0–26)	(0–18)	(0–3)	(0–26)
**Bac303**	Control	8.4	6.8	1.4	0.2
		(0–225)	(0–88)	(0–30)	(0–11)
	IBD	22.1^a^^,^^b^	14.9	1.1	0.1^a^^,^^b^
		(0–1082)	(0–163)	(0–55)	(0–3)
	Neoplasia	91.7^a^^,^^b^	14.9	2.2	0.4^b^
		(0–1872)	(0–104)	(0–31)	(0–14)
	GC	16.3^b^	7.1	0	0
		(0–210)	(0–56)		
**Hel717**	Control	0.4	7.2	0	0
		(0–30)	(0–177)		
	IBD	0	0^a^^,^^b^	0	0
	Neoplasia	0	0.3^a^^,^^b^	0	0
			(0–13)		
	GC	0	0	0	0

Data expressed as mean and range.

^a^ significant (*P* < 0.05) difference between control and CE dogs.

^b^ significant (*P* < 0.05) difference between CE groups.

**Fig 2:**
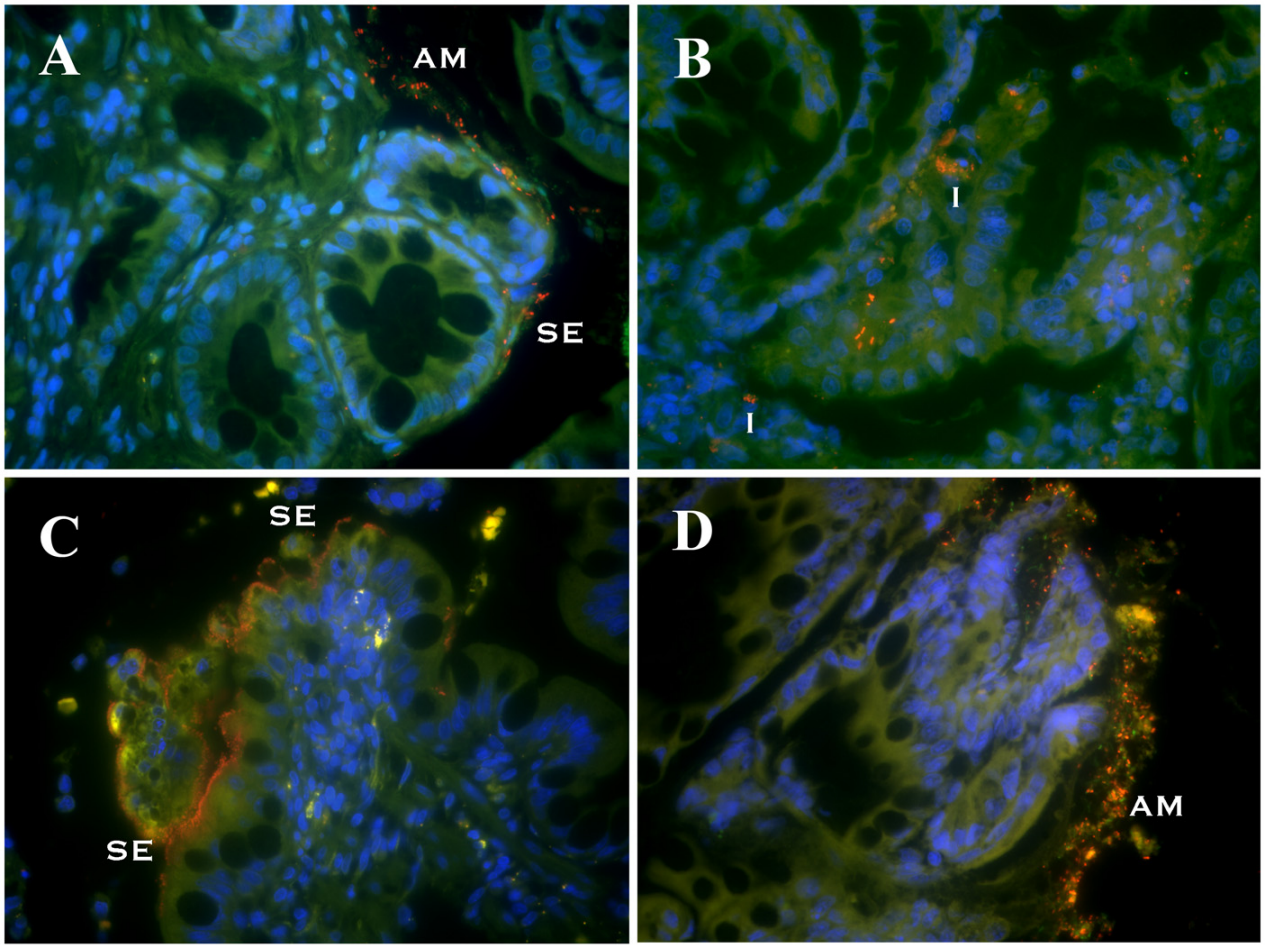
FISH of canine endoscopic biopsies. Triple color FISH identifies bacterial organisms within different mucosal compartments of endoscopic ileal and colonic biopsies obtained from dogs with CE. Panel A = colon biopsy hybridized with Cy3-Erec482; Panel B = colon biopsy hybridized with Cy3-Ec (*E*. *coli*); Panel C = ileum hybridized with Cy3-Ebac1790; and Panel D = ileum hybridized with Cy3-Bac303. All other bacteria that hybridize exclusively with the universal probe (Eub338-FITC) appear green. DAPI-stained colonic mucosa with goblet cells appears blue. AM = adherent mucus; SE = attached to surface epithelia; I = invasive within mucosa.

**Fig 3:**
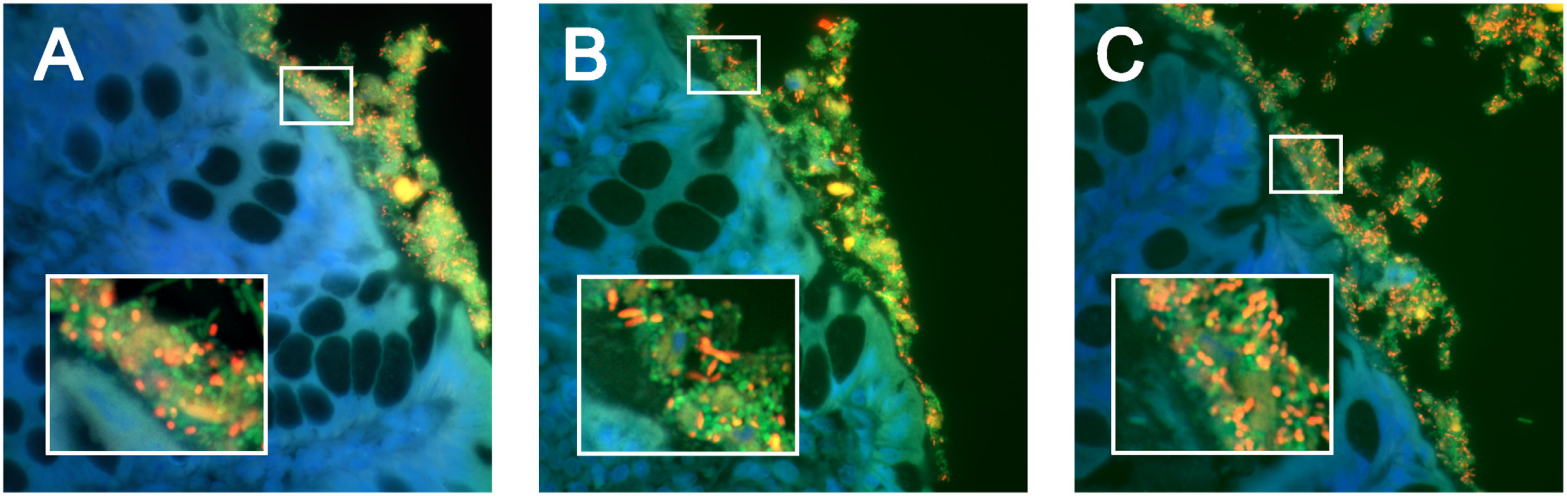
FISH of canine endoscopic biopsies. Triple color FISH identifies bacterial organisms present in biofilms adherent to the colonic epithelia in dogs with inflammatory bowel disease (A), granulomatous colitis (B), and colorectal cancer (C). *Bacteroides* (Bac303-Cy3 probe–panel A), *E*. *coli* (Ec-Cy3 probe–panel B), and *Eubacterium rectale* (Erec482-Cy3 –panel C) populations appear orange against green and blue backgrounds. All other bacteria that hybridize exclusively with the universal probe (Eub338-FITC) appear green. DAPI-stained colonic mucosa with goblet cells appears blue.

The spatial distribution of attaching bacteria in dogs with CE was significantly (*P* < 0.05) different from healthy dogs, with higher numbers of total bacteria (Eub338), *Eubacterium rectale* (Erec482), *Enterobacteriaceae* (Ebac1790), and *E*. *coli* (Ec) detected within colonic tissues (Fig 2). Some of these mucosal populations were characterized as biofilms of bacteria adherent to mucosal epithelia (Fig 3). Dogs with colonic neoplasia had increased (*P* < 0.05) numbers of attaching bacteria hybridizing against probes Eub338, Erec482, and Ec. Similarly, increased (*P* < 0.05) numbers of attaching bacteria hybridizing to probes Eub338 and Erec482 were observed in ileal biopsies of CE dogs. Within the CE group, dogs with intestinal neoplasia had the greatest (*P* < 0.05) number of attaching *E*. *coli* (Ec) and *Enterobacteriaceae* (Ebac1790) bacteria. Differences in the number of invasive bacteria within colonic mucosa included increased total bacteria (Eub338) but decreased *Bacteroides* spp (Bac303) in IBD dogs relative to healthy dogs; increased *Enterobacteriaceae* (Ebac1790) and *E*. *coli (*Ec) in GC dogs versus other CE dog groups; and increased *Bacteroides* spp (Bac303) in dogs with neoplasia versus other CE dog groups, *P* < 0.05 for all comparisons. In contrast, only *E*. *coli* (Ec) invasion (*P* < 0.05) was observed in ileal biopsies of GC dogs.

### Correlation between mucosal microbiota and inflammatory indices

The correlation between mucosal bacteria and inflammatory indices (CIBDAI score and histopathologic severity) was investigated to determine whether mucosal association might serve as a potential marker of disease activity in dogs with IBD and GC. Spearman’s rank correlation coefficient showed no significant correlation between mucosal bacterial sub-populations in the ileum and colon and the CIBDAI or histopathology score in IBD dogs and dogs with GC. However, there was a significant positive correlation (*P* = 0.026) between the number of total bacteria (probe Eub338) attached to the colonic mucosa and the CIBDAI score in IBD dogs.

## Discussion

The pathogenesis of CE in dogs is believed to result from dysregulated host responses directed against intestinal antigens, such as food antigens and bacteria [21–23]. In support of this notion, culture-independent investigations using gene clone libraries, polymerase chain reaction (PCR), and DNA isolation for microbial abundance have demonstrated imbalance of the microbiota in the diseased canine intestines [4–7,24]. Those data have largely been generated only from IBD dogs using duodenal specimens for comparative analysis of bacterial populations. In regards to the evaluation of the distal intestinal microbiota detailed investigations are clearly lacking. Only limited studies have incorporated FISH technology to investigate mucosal microorganisms in colonic endoscopic biopsies of dogs with CE [10,11]. In the present study, we report for the first time, the use of an array of FISH probes to visualize the composition and spatial organization of canine ileal and colonic mucosal microbiota in health and disease. Our results indicate that mucosal bacterial populations in the ileum and colon vary between different forms of CE in dogs.

Previous studies in humans have shown an association between GI signs, histopathologic inflammation, and microbial imbalances in the intestine, especially with IBD (ie, Crohn’s disease [CD] and ulcerative colitis [UC]) [25–28]. In general, these investigations have shown a relative decrease in the bacterial phyla *Firmicutes* and *Bacteroidetes*, while an increase in *Proteobacteria* and *Actinobacteria* were observed with mucosal inflammation. Moreover, a reduction in the diversity of *Clostridium* clusters XIVa and IV (ie, *Lachnospiraceae* and *C*. *coccoides* subgroups) are associated with IBD, suggesting that this bacterial group is important in disease pathogenesis, possibly through their production of short chain fatty acids (SCFA) [29,30]. Mucosally invasive *E*. *coli* is an especially important bacterium that appears enriched in some Crohn’s patients and linked to defects in innate immunity (ie, autophagy genes ATG16L1 and IRGM) which can promote and perpetuate intestinal inflammation [31,32].

While earlier studies have predominantly reported changes in fecal microbiota, there is less published information about the spatial organization of the mucosal microbiota seen with human IBD. Knowledge of host-microbiota interactions, in particular the role of attaching and invading bacteria, is important since an abnormal mucosal microbiota may interact more closely with the innate immune system to modulate gut health and disease. For example, Kleessen et al used FISH to study differences in bacterial populations residing on the mucosal surface and/or invading the terminal ileum and colonic tissues of human IBD patients [33]. Overall, more bacteria were detected on the mucosal surface of IBD patients versus non-IBD controls. Furthermore, bacterial invasion of the mucosa was evident in 83% of colonic specimens from UC patients and in 56% of the ileal and 25% of the colonic specimens from CD patients. In separate investigations, Swidsinski et al also used FISH evaluation of the mucosal microbiota in biopsy specimens from humans with CE, including IBD [34,35]. Their results indicated that IBD patients had high concentrations of mucosal bacteria and that an adherent mucosal biofilm composed mainly of *Bacteroides fragilis* is a prominent feature of IBD. Common characteristics of all 3 studies included increased mucosal concentrations of *Proteobacteria*, *Enterobacteriaceae*, and the *Bacteroides/Prevotella* cluster found in IBD patients.

The present study reveals that the ileal and colonic mucosal of healthy dogs and dogs with CE is predominantly colonized by bacteria localized to the free and adherent mucus compartments. There were marked differences in the composition of the mucosal microbiota of healthy versus CE dogs, with probes directed against *Eubacterium rectale*, *Bacteroides*, *Enterobacteriaceae* and *E*. *coli* significantly increased compared to these same bacterial sub-populations observed in healthy dogs. The group of IBD dogs, in particular, had increased mucosal concentrations of *Bacteroides* and *Enterobacteriaceae* in both ileal and colonic biopsy specimens while *Clostridia* and *E*. *coli* were increased in ileal and colonic mucus, respectively. An important observation was the spatial re-distribution of *Enterobacteriaceae* and *E*. *coli* bacteria attached to the surface epithelia and invasive within the mucosa of IBD dogs. This selective enrichment o*f Enterobacteriaceae* and *E*. *coli* in IBD tissues parallels reports of microbial shifts involving the fecal and intestinal mucosal microbiota of other canine cohorts (as determined by 454-pyrosequencing, gene clone libraries), cats, and humans with IBD [4–7,13,26,29–31].

Dogs with granulomatous colitis (GC) had severe clinical disease in spite of relatively low CIBDAI scores. This fact is related to the derivation of CIBDAI relative to abnormalities involving colonic function alone; that is, only changes in stool character (ie, semi-solid feces, mucus, and bright red blood) and frequency of defecation are observed most often [3]. The concentrations of bacteria hybridizing with Eub338, Erec482, Ebac1790, and Ec probes were significantly increased in the ileal and colonic mucus of GC dogs. All boxer dogs in this CE group had increased numbers of attaching/invasive *E*. *coli* (AIEC) bacteria occurring as clusters of organisms within inflamed ileal and colonic mucosa. Of interest is the previous finding that the strains of *E*. *coli* isolated from Boxer dogs with GC have high phylogenetic resemblance to *E*. *coli* associated with Crohn’s disease (ileitis) in humans [36]. Mutations in genes regulating autophagy and bacterial clearance (ie, ATG16L1 and NCF2 genes) are thought to contribute to enhanced host susceptibility to AIEC infection in humans and dogs, respectively [23,28,37].

Intestinal neoplasia involving the colorectal and ileal mucosa comprised a group of older dogs with focal masses involving the descending colon and/or rectum (n = 7, all with adenocarcinoma) or having patchy or diffuse infiltrative disease involving the small and large intestines and diagnosed with either adenocarcinoma (n = 2) or lymphoma (n = 3). In general, the intestinal tissues of cancer dogs contained the highest numbers of mucosal bacteria hybridizing to probes Eub338, Erec482, Ebac1790, Ec, and Bac303 as compared to tissues obtained from healthy dogs and dogs with non-cancer CE. This was especially true within the free/adherent mucus compartments of both colonic and ileal biopsies. A similar trend was observed for increased mucosal bacteria to be found attaching to surface epithelia or invading within colonic (probes Eub338, Erec482, Ec and Bac303) and ileal (probes Ebac1790 and Ec) biopsies of cancer dogs versus dogs with other forms of CE.

The intestinal microbiota is increasingly linked with colorectal cancer (CRC) in humans. Recent studies indicate that *Fusobacterium* spp. generate a pro-inflammatory microenvironment that is conducive to CRC progression likely through recruitment of tumor-infiltrating immune cells [38]. Moreover, microbial mucosal shifts consisting of tumor enrichment with both *Fusobacterium nucleatum* and *Enterobacteriaceae* have been observed in CRC tumors [39,40], while invasive polymicrobial mucosal biofilms may serve as a distinct feature of more proximal CRC in humans [41]. Whether increased mucosal concentrations of *Fusobacteria* (not assessed in the present study) or other bacterial species are causally associated with mucosal inflammation and progression to CRC in dogs will require further study.

There was no statistical correlation between mucosal bacterial sub-populations in the ileum and colon and the CIBDAI or histopathology score in IBD dogs and dogs with GC. This finding may have been due to the fact that we evaluated only the number of attaching/invading bacteria, versus mucus-laden bacteria, in canine cohorts having mucosal inflammation. This could also be influenced by the small study size (especially in GC dogs), the contribution from non-bacterial factors mediating GI disease (ie, local immune response, motility disturbances), and/or the difficulties inherent in the histopathologic interpretation of endoscopic biopsies [42]. In spite of these factors, we did observe a significant positive correlation between the number of total bacteria attached to the colonic mucosa and the CIBDAI score in IBD dogs having moderate-to-severe disease severity.

There are some potential limitations in this study. It is possible that some dogs (n = 3) may have received several days of antibiotics within 3 weeks of GI referral and diagnostic evaluation for CE. In these instances, referring veterinarians had been treating animals short-term for routine non-GI related conditions including urinary tract infection (n = 1), pyoderma (n = 1), and bacterial-mediated otitis externa (n = 1). Since FISH with rRNA-targeted probes is dependent on the rRNA content (ie, metabolic activity) of individual bacteria, it is possible that previous antibiotic administration may have reduced the proportion of some mucosal bacteria accessible by FISH [35]. All dogs in the present study received routine colonic cleansing prior to collection of ileal and colonic mucosal biopsies. It is possible that dogs cleansed by colonic electrolyte lavage and enemas might have had disrupted mucus compartments characterized by reduced bacterial populations. However, we have previously investigated bacterial numbers by FISH in pilot studies using untreated colonic specimens and found that mucus compartments do not differ appreciably between purged vs. non-purged dogs (AEJ, unpublished observation). These findings are in accordance with results in human IBD where differences in the mucus barrier of intestinal bacteria were not observed between patients prepared by oral electrolyte lavage or enema versus patients which did not receive colonic cleansing [43]. The precise mechanism(s) by which CE may alter the intestinal microbiota are beyond the scope of this study. It is possible that each of the disease conditions may have alterations in mucosal innate and adaptive immune responses, particularly in release of REG-III, alpha and beta defensins or cathelcidins, mucin production, increased intestinal permeability, and/or altered mucosal regulatory/cytotoxic T cell and dendritic cell activity [44]. It is also possible that the various canine CE affect nutrient digestion and absorption, thereby altering the micronutrients available for the intestinal microflora.

Regarding the effect of formalin fixation [35] on integrity of the intestinal mucus layer (versus Carnoy’s fixative), our experiences using FISH in multiple species suggest that this is not a problem and that the mucus layer remains largely intact even with routine tissue processing. We have previously demonstrated the presence of an intact and largely continuous epithelial mucus layer (via Alcian blue stain) in formalin-fixed endoscopic ileal and colonic specimens of study dogs (data not shown). Moreover, other investigators have shown the utility of FISH used on formalin-fixed biopsy specimens obtained from the GI tract of companion animals [14,45] and humans [31,34,46–48].

Another potential factor impacting quantification of mucosal bacteria might be mechanical artifacts associated with tissue processing (microtome cutting) and/or non-intended wash of biopsy specimens by formalin during transport to the pathology laboratory [43]. Our previous experiences have allowed us to readily identify these tissue artifacts (in companion animals and mice) and to avoid these areas, if present, when performing mucosal bacterial counts. Finally, gut microbial populations may potentially vary by age, gender, breed, and dietary consumption. Our own studies, evaluating the potential impact of age, body weight, and/or diet have not identified any associations of microbial abundances with these variables in dogs to date [5,49,50].

In summary, the present study indicates that dogs with CE exhibit imbalances in the composition and structure of their mucosal microbiota. Moreover, we show that mucosal bacterial populations in the ileum and colon vary between the different forms of canine CE, and that this is especially true for dogs with IBD or intestinal neoplasia where intestinal tissues are selectively enriched in mucosal populations of *Enterobacteriaceae* and *E*. *coli*. These spatial, segment-specific structure and differential response of select bacterial groups to intestinal inflammation may be pivotal regarding the functional consequences of these alterations in the pathogenesis of canine CE [49].

## Abbreviations

FISH: fluorescence in situ hybridization
IBD: idiopathic inflammatory bowel disease
GC: granulomatous colitis
CIBDAI: canine inflammatory bowel disease activity index
GI: gastrointestinal
16S rRNA: 16S ribosomal RNA
